# Antimicrobial resistance from a One Health perspective in Zambia: a systematic review

**DOI:** 10.1186/s13756-023-01224-0

**Published:** 2023-03-03

**Authors:** Avis A. Nowbuth, Akwi W. Asombang, Nkengeh N. Tazinkeng, Opeoluwa Y. Makinde, Lincoln R. Sheets

**Affiliations:** 1 Lusaka Apex Medical University, Lusaka, Zambia; 2 School of Medicine, University of Missouri-Columbia, Columbia, MO USA; 3 Pan-African Organization for Health, Education and Research, Manchester, USA; 4 Present Address: Massachusetts General Hospital, Boston, USA; 5 Central Administration University of Buea, Buea, Cameroon; 6 Obafemi Awolowo University, Ife, Osun State Nigeria

**Keywords:** Antimicrobial resistance, One Health, Zambia, Review

## Abstract

**Background:**

Antimicrobial resistance (AMR) is widely acknowledged as a global health problem, yet its extent is not well evaluated, especially in low-middle income countries. It is challenging to promote policies without focusing on healthcare systems at a local level, therefore a baseline assessment of the AMR occurrence is a priority. This study aimed to look at published papers relating to the availability of AMR data in Zambia as a means of establishing an overview of the situation, to help inform future decisions.

**Methods:**

PubMed, Cochrane Libraries, Medical Journal of Zambia and African Journals Online databases were searched from inception to April 2021 for articles published in English in accordance with the PRISMA guidelines. Retrieval and screening of article was done using a structured search protocol with strict inclusion/exclusion criteria.

**Results:**

A total of 716 articles were retrieved, of which 25 articles met inclusion criteria for final analysis. AMR data was not available for six of the ten provinces of Zambia. Twenty-one different isolates from the human health, animal health and environmental health sectors were tested against 36 antimicrobial agents, across 13 classes of antibiotics. All the studies showed a degree of resistance to more than one class of antimicrobials. Majority of the studies focused on antibiotics, with only three studies (12%) highlighting antiretroviral resistance. Antitubercular drugs were addressed in only five studies (20%). No studies focused on antifungals. The most common organisms tested, across all three sectors, were *Staphylococcus aureus*, with a diverse range of resistance patterns found; followed by *Escherichia coli* with a high resistance rate found to cephalosporins (24–100%) and fluoroquinolones (20–100%).

**Conclusions:**

This review highlights three important findings. Firstly, AMR is understudied in Zambia. Secondly, the level of resistance to commonly prescribed antibiotics is significant across the human, animal, and environmental sectors. Thirdly, this review suggests that improved standardization of antimicrobial susceptibility testing in Zambia could help to better delineate AMR patterns, allow comparisons across different locations and tracking of AMR evolution over time.

**Supplementary Information:**

The online version contains supplementary material available at 10.1186/s13756-023-01224-0.

## Background

The SARS-COV-2 pandemic has highlighted the importance of implementing working systems at a local level to mitigate and prevent spread of infectious diseases. Antimicrobial resistance (AMR) has been highlighted by the World Health Organization (WHO) as a prominent threat to global health [1]. There is specific concern of low-middle income countries (LMICs) where there is poor surveillance, poor diagnosis measure and a lack of guidelines indicating therapy procedures [2]. A regional and national understanding of AMR is needed to improve human health, animal health and agricultural productivity per country [3]. A 2022 study highlights that bacterial AMR is the most prevalent cause of death related to drug resistance [4]. Given the global importance of bacterial AMR, there is an urgent need to highlight the clinically relevant resistance related to bacteria, especially in LMICs [4]. LMICs have unique socioeconomic and cultural settings that challenge the strategies from policy makers on the world stage [5], as a result of this, antimicrobial stewardship strategies must be tailored specifically from the ground level. In many LMICs, the use of antimicrobials for treatment remain undocumented and unregulated [6].

The One Health Approach is the ‘collaborative effort of multiple disciplines – working locally, nationally, and globally – to attain optimal health for people, animals and our environment…’ [7] and recognizes that there is a link between these three domains. The WHO has stated that the One Health approach is critical to addressing health threats across all three interfaces [8]. The concept focuses on the consequences, responses and actions across the human-animal-environment sectors highlighting the importance of balance and interconnectedness. A solo approach only focusing on health education will not lead to effective results because these three sources are interrelated. AMR understanding and control should be approached ideally from a One Health perspective since resistance can arise in the human, animal or the environment and spread from one to another [9].

A report was developed by the Zambian National Public Health Institute (ZNHPI) and the Centre for Science and Environment (CSE) India highlighting the need to prepare a surveillance system for Zambia [10]. This report mentions the need for interventions, however, does not reflect the rate of AMR data and studies in Zambia to date. Zambia faces a generalized HIV epidemic, with most deaths resulting from opportunistic infections. Malaria is considered to be the main cause for hospitalization, and the biggest contributor to morbidity and mortality rates [10, 11]. There have also been outbreaks of cholera, meningococcal meningitis, pneumonia, and typhoid in Zambia [10]. Standard infections are becoming increasingly difficult to treat with standard first-line antibiotics because of AMR, leading to the necessary use of newer, more targeted, but also more expensive antibiotics [12, 13].

A consolidated approach is needed to address the complexity and scale of the problem which includes incorporating the various fields of governance and policy makers [14]. When an overview of antimicrobial practice is known, more tailored implementations and control measures can be planned; and understood. Unique insight into antibiotic prescribing, ideas about AMR, and insights into use of antimicrobials in the animal and agriculture sectors. We conducted a systematic review assessing the prevalence of AMR in Zambia as a means of establishing an overview of the situation, to help inform future decisions as there are currently no guidelines in place to monitor the use of antimicrobials, nor is there standardized testing procedures in place, to date. This review will assist in creating a more tailored approach in implementing interventions by highlighting the levels of resistance and lack of data that need to be addressed across all sectors.

## Methods

### Search strategy

**Fig. 1:**
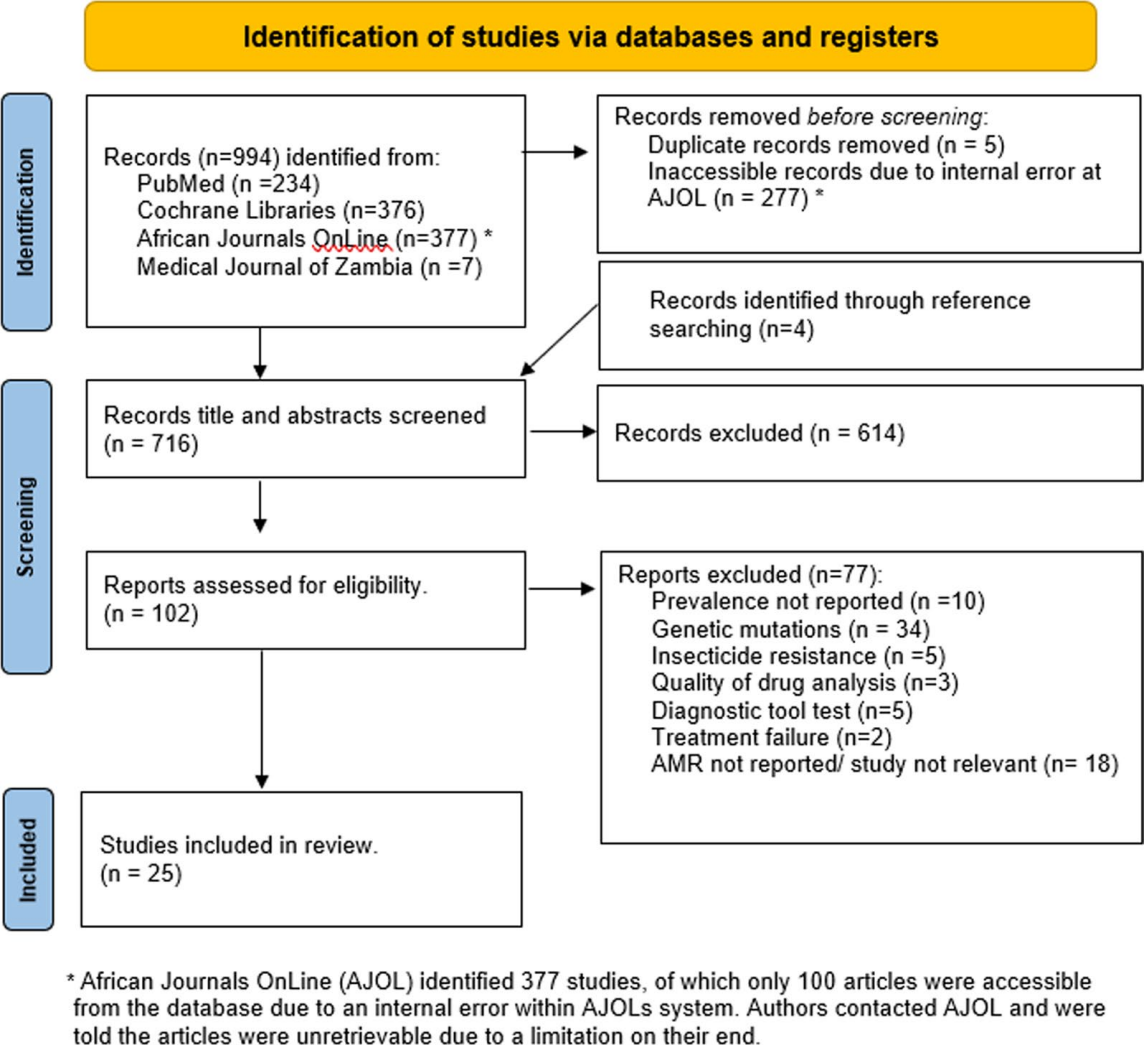
PRISMA flow-chart illustrating the study selection process on antimicrobial resistance in Zambia

A systematic review was performed in accordance with PRISMA (Preferred Reporting Items for Systematic Reviews and Meta-Analysis) guidelines (Fig. 1) [15]. The search terms [(*multiresistant* OR *multi-drug resistant* OR *antimicrobial resistance* OR *drug resistance* OR *bacterial resistance*) AND (*Zambia*)] were used to identify relevant literature from Cochrane Libraries, PubMed, Medical Journal of Zambia and African Journals Online databases. Various spellings of the search terms were considered. A total of 994 articles were identified from the four databases that were searched using Boolean search strategies to obtain English articles relating to AMR and Zambia. No limitation on publication dates were set. Literature search began in March 2021, with an update on April 30, 2021. Reference list of relevant articles were checked for additional titles for inclusion in the review. No limitation was set on the bacteria in animal and human health sectors.

### Inclusion and exclusion criteria

Full-text articles on the prevalence of antibiotic resistance among clinical pathogenic bacteria isolated from humans (inpatients, outpatients, healthy volunteers), animals (avian, cattle, sheep, swine, and fish) and environment (non-healthcare: water, markets, outbreak studies; and healthcare: hospital surfaces, health care tools,) in Zambia were used for the review. Publications were initially screened independently by three reviewers (AAN, NNT, OYM) to determine eligibility. Articles were reviewed by at least two reviewers and disagreements were resolved by a third author. Studies related to human sector included both adult and pediatric populations, inpatients and outpatients, and healthy volunteers in institutions such as prisons and schools. There were no limitations on the disease or microorganism tested. Publications identified through the literature search that reported AMR in human, animal, and environment but that did not report prevalence data were not included; specific site of genetic mutations resulting in AMR were not included. Studies that mentioned insecticide resistance, assessed the quality of the drug (drug composition, generic compounds and imported medications compared to local medications) were excluded as the studies did not mention resistance rates. Studies that tested the most appropriate diagnostic testing tools were excluded. Studies that made mention of compliance of chronic medicines such as in HIV, and treatment failures were rejected. Any publication that did not report data on AMR were considered not relevant and were excluded.

### Data extraction

Data was extracted from each study using a database developed by AAN for the purpose of this review using Microsoft Office 365: Excel. The data extraction was independently done by AAN and verified by co-authors NNT and OYM. Articles that met the inclusion criteria and reported prevalence data for AMR were included in the systematic review.

Information extracted included article information (first author, year of publication, duration of study, location, and specific sites), and study design (samples size, cross-sectional design, or longitudinal study). The specific information extracted for the human sector considered: category of patients (in- or out-patients, healthy persons and reason admitted if inpatient), and type of samples extracted (pus, blood, throat swabs, stool, nasal swabs, urine, vaginal or wound swabs). Regarding the animal sector: species, number of sites (including farms, sanctuaries, and veterinary sites), number of animals sampled in analysis, sample type (feces, meat, milk, blood), sampling point (farm, slaughterhouse, or retail market) were extracted. The environmental/agricultural sector included information of interest such as water, outbreak studies, vegetables, markets, clinic/hospital surfaces and medical tools were considered. Articles that studied more than one sector were classified as a One Health paper and extracted to the subsections mentioned above. The type of organism, organism numbers, antibiotics tested, and interpretation of the findings was extracted into each of the abovementioned category. The samples that were studied had to have undergone a laboratory procedure in which the type of microorganism was identified, and prevalence of antibiotic-resistant bacteria was taken into consideration for the human, animal, and environment articles. Specific site of genetic mutations was noted, highlighting the degree of resistance a microorganism has, however the specific sites were not included in this systematic review.

### Data analysis

Articles were characterized based on Zambia as a geographic location (including the region and specific site if this information was available; if the study included other countries, specific information regarding Zambia was extracted if available), the type of antimicrobial resistance described in the study (antibacterial, antiviral, antifungal or antimalarial), context of the study (human, animal or environmental; or a combination of any of the sectors i.e. One Health), study design, and outcome of the studies (specifically prevalence rate of antimicrobial resistance). Meta-analysis was not conducted due to the diversity of the study types and identified data, and therefore present descriptive findings. Visualizations were performed using Microsoft Office 365.

## Results

### Data

The initial search of the online databases identified a total of 994 publications (PubMed (n = 234), Cochrane Libraries (n = 376), African Journals Online (AJOL) (n = 377) and Medical Journal of Zambia (MJZ) (n = 7)) from inception of database to April 2021. A total of 5 duplicates were removed. Additional 4 records were retrieved after screening references. The African Journals Online identified 377 studies, of which only 100 articles were accessible from the database due to an internal error within AJOLs system. A total of 716 studies were screened for eligibility based on the title and abstract contents. Overall, 614 articles were excluded for non-relevance to this systematic review. 102 full-text articles were assessed for eligibility with 25 articles meeting the inclusion criteria for the study (Fig. 1). Table 1 summarizes the characteristics of the analyzed articles; a full list of the included articles and breakdown of findings is provided in Table 2 and further details of specific antimicrobial results is found in Additional file 1/Supplementary Data.

**Table 1:** Summary of the articles included in the systematic review

Resistance type	Number of Articles	AMR context (Samples studied)				Output	
		Human (H)	Animal (A)	Environmental (E)	One Health (AHE)	Policy	Surveillance
*a*							
Antibacterial	16	9 (+ 2)	2 (+ 2)	2 (+ 2)	3 (AE, HE, HA)	0	16
Antimalarial	1	1	0	0	0	0	1
Antiviral	3	3	0	0	0	0	3
Antitubercular	5	5	0	0	0	0	5
Antifungal	0	0	0	0	0	0	0
Total	25	18 (20)	2 (4)	2 (4)	3	0	25

	Pathology in human samples	n (%) (n = 20)	Study
*b*			
1	Breast abscess	1 (5)	Kapatamoyo et al. [16]
2	Cholera	1 (5)	Mwape et al. [17]
3	Chronic suppurative otitis media	1 (5)	Matundwelo and Mwansasu [18]
4	Diarrheal disease	2 (10)	Mainda et al. [19, 20], Chiyangi et al. [21]
5	HIV	4 (20)	Gill et al. [22], Bennett et al. [23], Inzaule et al. [24], Miti et al. [25]
6	Hospital acquired infections	1 (5)	Chanda et al. [26]
7	Malaria	1 (5)	Bijl et al. [27]
8	Neonatal sepsis	1 (5)	Kabwe et al. [28]
9	Not mentioned	2 (10)	Ziwa et al. [29], Nagelkerke et al. [30]
10	Tuberculosis	5 (25)	Mulenga et al. [31], Habeenzu et al. [32], Kapata et al. [33, 34], Masenga et al. [35], Kapata et al. [33, 34]
11	Typhoid	1 (5)	Hendriksen et al. [36]

	Samples	Human studies	Animal studies	Total Studies (A + E)	Total %
*c*					
1	Blood	7		7	26
2	Sputum	5		5	19
3	Stool	4		4	15
4	Wound Swab	3	1	4	15
5	Aspirates	1		1	4
6	Nasal swab	1	1	2	7
7	Rectal swab	1		1	4
8	Urine	1		1	4
9	Oral swab	1	1	2	7

**Table 2:** Characteristics of articles included in the systematic review

Type of study	Author	Year published	Location	Human		`Animal			Environmental	
				Sample SOURCE	Microorganism	Species	Sample type	Microorganism	Sample Source	Microorganism
One Health	Mainda et al. [20]	2019	Lusaka, LP	Stool	*E. coli*	Cattle		*E. coli*		
	Ziwa et al. [29]	2018	Lusaka, LP	Wound swabs	*S. aureus* *K. pneumoniae*				Hydrotherapy Bathtub	*S. aureus* *K. pneumoniae*
	Youn et al. [37]	2014	Lusaka, LP			Dogs Cats	Wound swabs	*S. aureus* *S. pseudintermedius*	Door handles, Desk Examination table, Medical Devices, Scale, Floor, Tray surface, Operation table	*S. aureus* *S. pseudintermedius*
Environ	Songe et al. [38]	2016	Mongu, WP						Mongu Fish Markets: Flies	*E. coli*
	Mwamungule et al. [39]	2015	Lusaka, LP						Doctors White Coats	*S. aureus* *K. pneumoniae*
Animal	Mainda et al. [19]	2015	Zambia			Cattle		*E. coli*		
	Schaumburg et al. [40]	2012	Chingola, CoP			Chimpanzees	Oral swabs Nasal swabs	*S. aureus*		
Human	Kapatamoyo et al. [16]	2010	Lusaka, LP	Pus Aspirates	*S. aureus*					
	Matundwelo and Mwansasu [18]	2016	Ndola, CoP	Pus Swab	*S. aureus* *P. vulgaris*					
	Miti et al. [25]	2020	Ndola, CoP	Blood	*HIV*					
	Nagelkerke et al. [30]	2017	Eastern EP	Nasal Swab	*S. aureus*					
				Rectal Swab	*Enterobacteriaceae*					
	Inzaule et al. [24]	2020	Zambia	Blood	*HIV*					
	Bennett et al. [23]	2020	Lusaka, LP	Dried Blood Spots	*HIV*					
	Mwape et al. [17]	2020	Lusaka, LP	Stool	*V. cholerae*					
	Kapata et al. [34]	2015	Lusaka, LP	Sputum	*M. tuberculosis*					
	Hendriksen et al. [36]	2014	Lusaka, LP	Stool	*S. typhi*					
				Blood						
	Masenga et al. [35]	2017	Livingstone, SP	Sputum	*M. tuberculosis*					
	Kapata et al. [33]	2013	Lusaka, LP		*M. tuberculosis*					
	Mulenga et al. [31]	2010	Ndola, CoP	Sputum	*M. tuberculosis*					
	Bijl et al. [27]	2000	Kaoma, WP	Blood	*P. falciparum*					
	Chiyangi et al. [21]	2017	Lusaka, LP	Stool	*V. cholerae NTS E. coli, S.Typhi S.paratyphi B, Shigella flexinari, Shigella dysenteriae, Shigella boydii, Campylobacter jejuni*					
	Habeenzu et al. [32]	2007	Zambia	Sputum	*M. tuberculosis*					
	Kabwe et al. [28]	2016	Lusaka, LP	Blood	*Klebsiella sp, S. aureus, E.coli*					
	Chanda et al. [26]	2019	Ndola, CoP	Blood, Urine, Wound swabs	*E. coli, Citrobacter species, Coliform, Streptococcus Enterobacteriaceae, S. aureus, coan Staphylococci, Pseudomonas sp.*					
					*Proteus species*					
					*Klebsiella sp.*					
	Gill et al. [22]	2008	Ndola, CoP	Nasal Swabs	*S. pneumoniae*					

### Study characteristics

A total of 18 studies reported on the outcome of AMR in humans, two reported on the outcome of AMR in animals and two on the environment (Table 1a). Three studies used a One Health approach; with one study reporting outcomes of AMR in both human and animal sectors, while one study reporting on the outcome of AMR in animals and environmental sectors, one study reflected on the outcomes of AMR within the human and environmental sector (Table 1a and Table 2). Across the sectors, 21 (84%) of studies were focused on antibacterial, with 5 studies specific for antitubercular drugs, and 3 studies focused on antiretrovirals. The antimicrobials that underwent surveillance were antibiotics (antibiotic classes were aminoglycosides, cephalosporins, penicillin, sulfonamides, fluoroquinolones, macrolides, tetracyclines, chloramphenicol and glycopeptides), antivirals (nucleoside reverse transcriptase inhibitors (NRTI), non-nucleoside reverse transcriptase inhibitors (NNRTIs) and protease inhibitors (PIs). Only one paper reported data on antimalarials. No antifungals were surveilled. All the studies relayed information about surveillance, highlighting zero studies on policy making.

**Fig. 2:**
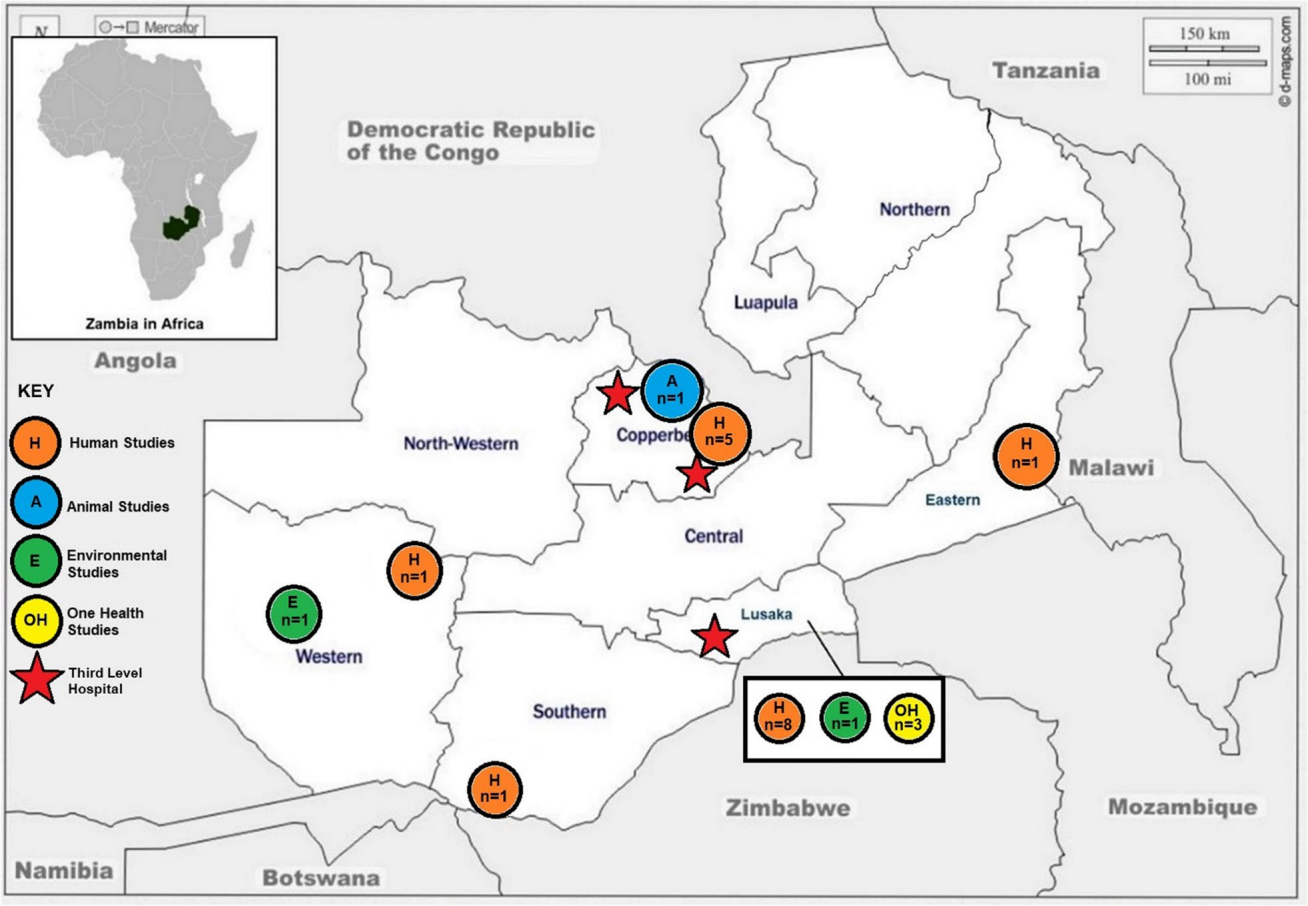
Map of Zambia showing study sites and number of articles used in the review

**Fig. 3:**
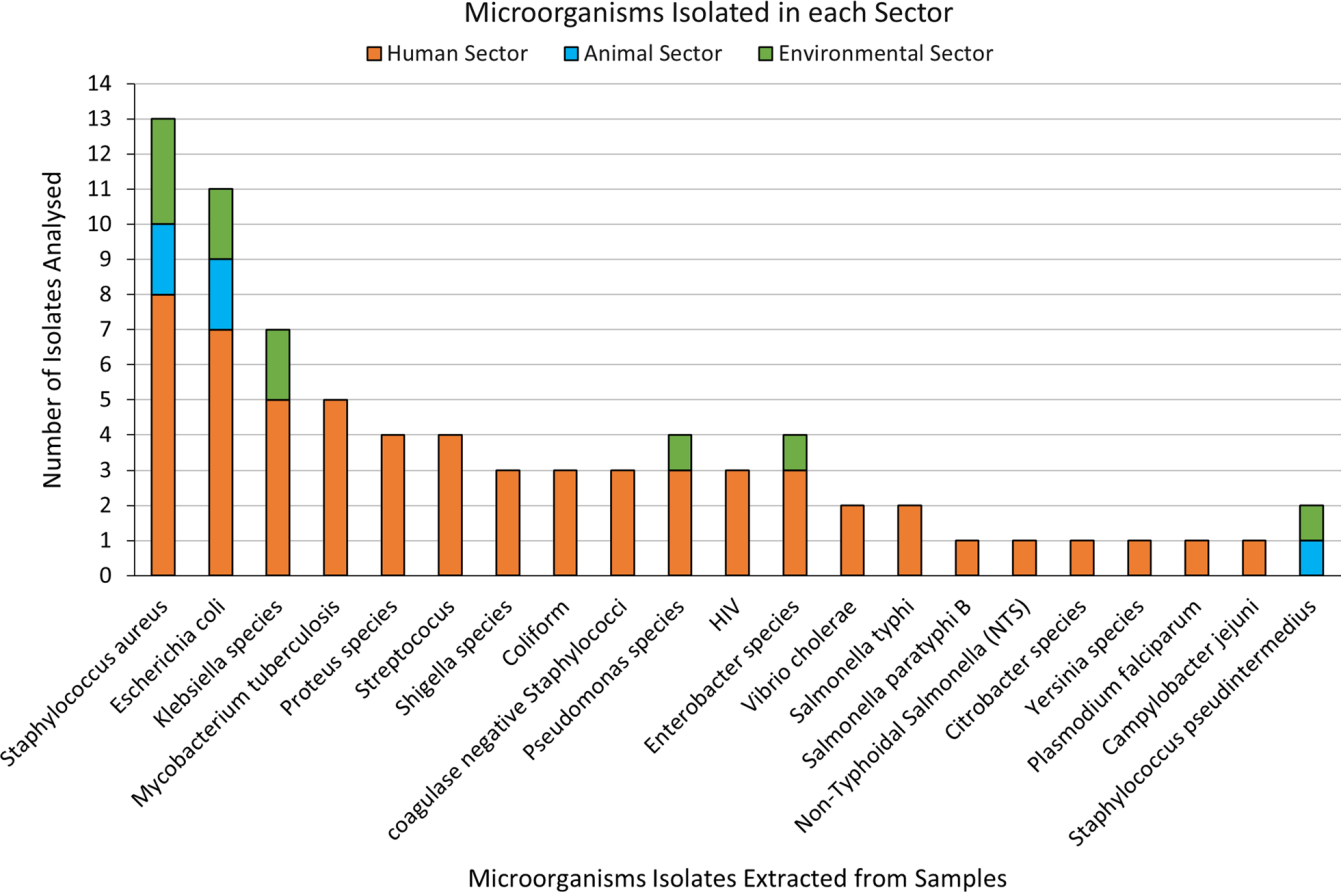
Microorganisms isolated across human, animal, and environmental sectors in Zambia

The largest number of studies originated from Lusaka Province (n = 12), followed by Copperbelt Province (n = 6), Western Province (n = 2), Southern Province (n = 1), Eastern Province (n = 1), while three studies mentioned multiple sites within the regions of the country (Fig. 2.) however, it was noted that several provinces had no studies conducted. From these studies, there were multiple samples taken, and multiple microorganisms sampled and tested (Table 2). These studies investigated isolates from diarrheal diseases (2; 10%), breast abscesses (1; 5%), chronic supportive otitis media (1; 5%), hospital acquired infections (1; 5%), malaria (1; 5%), neonatal sepsis (1; 5%), typhoid (1; 5%), HIV (4; 20%), and tuberculosis (4, 25%) (Table 1b). Overall, 27 samples were reported from the human and animal studies, the most abundant being blood (7; 26%) followed by sputum samples (5; 19%), stool samples (4; 15%), wound swabs (4; 15%), nasal swabs (2; 7%), aspirates (1; 4%), rectal swabs (1; 4%), urine samples (1; 4%) and oral swabs (2; 7%) (Table 1c) Two animal studies did not provide the source of samples for culture. Samples from the environmental sector were obtained from fish markets, veterinary hospital surfaces, healthcare white coats and the hydrotherapy bathtub of the burns unit at the Department of Surgery (Table 2). Twenty-one types of pathogens across all sectors were isolated from samples with the most common organisms isolated being *Staphylococcus aureus* (13; 17%) followed by *Escherichia coli* (11; 14%), *Klebsiella species* (7; 9%), *Mycobacterium tuberculosis* (5; 7%), *Proteus species* (4; 5%), *Streptococcus species* (4; 5%), *Pseudomonas species* (4, 5%), *Enterobacter species* (4; 5%), *Coliform* (3; 4%), *Shigella species* (3; 4%), *coagulase negative Staphylococci* (3; 4%), HIV (3; 4%), *Vibrio cholerae* (2; 3%), *Salmonella Typhi* (2; 3%), *Salmonella paratyphi* B (1; 1%), *Non-Typhoidal Salmonella* (NTS) (1; 1%), *Citrobacter species* (1; 1%), *Yersinia species* (1; 1%), *Plasmodium falciparum* (1; 1%), *Campylobacter jejuni* (1; 1%) and *Staphylococcus pseudointermedius* (2; 3%) as summarized in Fig. 3 and detailed in Table 2. The susceptibility of these isolates towards 36 antimicrobial agents across 13 classes of antibiotics was tested.

The animal, environmental and One Health studies focused on bacterial pathogens and resistance patterns. The most found pathogens isolated were *E. coli* and *S. aureus*. *Salmonella* was only studied in the human sector. The 25 included studies focused on resistance profiles of microbes. No articles included described the process or outcomes of AMR stewardship programs at a local level or focused on policy aspects of AMR prevention or management. No studies were conducted that relayed information about antifungal resistance.

## Discussion

Antimicrobial Resistance has been highlighted by the World Health Organization (WHO) as a global health threat that needs urgent intervention [13, 41]. Zambia has a National Action Plan (NAP), aimed to have been implemented by 2020 [10], however the effects of these interventions may be inaccurate without a baseline investigation. As an LMIC, there are multiple challenges Zambia must overcome, such as the unavailability of diagnostic tests, lack of microbiologists at teaching hospitals, lack of reagents or inadequate equipment within hospitals and laboratories. Availability of routine and research data on antimicrobial resistance is an important step in the development of local strategies to curb the global AMR crisis [42]. The One Health approach is a progressive development of multi-disciplinary action across the human health sector with animal and environmental health [43]. A report was developed by the ZNHPI and the CSE India highlighting the need to prepare a surveillance system for Zambia [10]. An integrated baseline using information collected from secondary research was developed with the hope to accurately implement a NAP. The report mentions commonly used antimicrobials, disease burdens and estimates the trends of AMR from secondary studies conducted in Zambia [10].

This current review describes published data on antimicrobial drug resistance from Zambia, revealing a high rate of resistance of microorganisms isolated in hospital settings, animal health and environment against typical antibiotics used in Zambia. Lusaka province had majority of the studies, with various provinces having no data on AMR, indicative that more AMR data is needed for several provinces to have a more complete understanding of the status of AMR in the country. A possible explanation for why studies are conducted in Lusaka could be because Lusaka has the highest population density, with the biggest of the teaching hospitals in Zambia located in this province. There are more resources, more sites, and greater ease to collaborate in a One Health approach in Lusaka, compared to the smaller, distanced cities in Zambia. A couple of articles mentioned Zambia in a multi-country study, however in these cases, the specific region or location of the study or sample collection was rarely mentioned, resulting in limitations of the study in findings specific to the Zambian context. A 2017 systematic review reported that about 42% of African countries do not have published studies on AMR [44], and the lack of information available in Zambia is evident. Many LMICs, such as Zambia, are resource limited. The allocation of resources is crucial hence an up-to-date baseline is needed to develop, coordinate and apply surveillance systems at all levels [10].

Many studies in sub-Saharan Africa focus on antibacterial resistance with few studies on antivirals and antifungals. Countries with high prevalence of HIV, TB and malaria had high numbers of studies on these diseases [10]; this does not align with our observations from Zambia. Despite Zambia having an HIV epidemic, only four studies made mention of HIV, with one study testing the resistance to opportunistic infections in HIV patients: *S. pneumoniae*. HIV prevalence in Zambia was estimated 11.3% among adults ages 15 to 49 as of 2018 [45]. This represents a substantial burden of disease, and it was surprising to identify only three studies focusing on prevalence to antiretroviral resistance in Zambia: particularly because of the emerging resistance to antiretroviral regimens across Africa [46]. Zambia has a high prevalence of tuberculosis with 455/100,000 cases recorded in a study in 2019 [47]. Despite the tuberculosis epidemic, only five studies showed specific resistance to antitubercular drugs. The detection of *M. bovis* in Zambia and LMICs is limited due to poor laboratory facilities and lack of trained personnel [47]. Bovine tuberculosis (BTB) has been reported in traditional cattle in Zambia, with a high prevalence of 49.8% within the Kafue basin region; while abattoirs in Namwala district found that 16.8% of cattle slaughtered were infected with BTB [47]. The spill-over effect from the animal sector to the human sector has not yet been established in Zambia. A recent study found that there is a need for routine laboratory surveillance and better case managements to prevent and limit multidrug resistant TB in Zamia [48]. In Copperbelt Province, Zambia, Monde et al. [48] shows that there is emergence of *Mycobacterium tuberculosis* complex which are resistant to one or more anti-tuberculosis drugs.

**Fig. 4:**
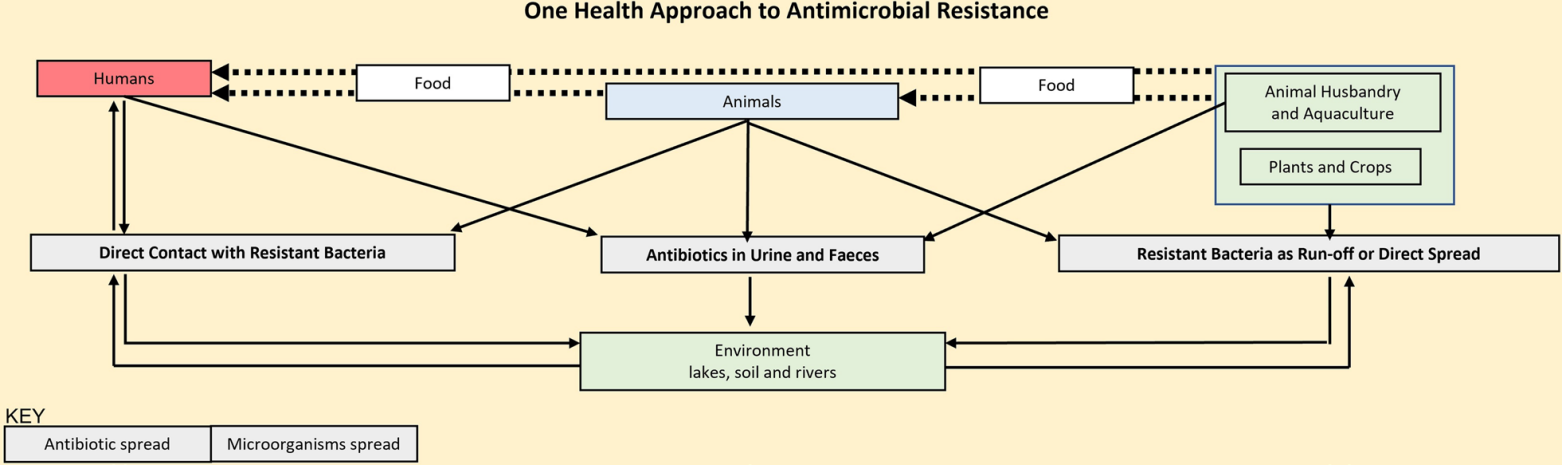
One Health approach to antimicrobial resistance

Most studies on AMR in Zambia focused on resistance in the human sector, with a handful of studies in the animal and environmental sectors, despite the known importance of the interconnectedness of all three sectors and the vital role they play in preventing and mitigating AMR. The One Health approach should assist and encourage future researchers to consider the methodologies that explicitly look at the interlink across human-animal-environment frameworks, specifically focusing on the zoonotic diseases that have the high potential of resulting in resistance to antimicrobials. With only three studies focusing on multiple sectors, yet these studies are on microorganisms that are not epidemiological microorganisms, it was noted that there are not enough studies using the ‘One Health’ approach in Zambia. More studies highlighting the spill-over effect will be needed to establish a better overview and a better way to combat AMR in Zambia. Understanding these elements and addressing them from the ground-level is necessary to change the modifiable interactions to reduce or interrupt the spread of resistance from the environment into clinical, and animal settings; and vice versa (Fig. 4). Figure 4 shows the relationship between the sectors, and the importance of recognizing this collaboration.

Numerous studies found resistance of more than 50% suggesting high resistance or possible sampling/testing errors. It was observed that studies are diverse in samples type, study design and identified data and no standardized tool was used across the studies. Antibiograms were not found in the systematic review, and the most recent antibiogram found in a ZNHPI report was from 2016 for only one hospital [10]. Recent communication with the laboratory mentioned a shortage of reagents hence the main third level hospital cannot conduct yearly antibiograms, as recommended by the Clinical and Laboratory Standards Institute (CLSI) [49].

Several studies showed a 100% resistance to commonly prescribed drugs in Zambia, as well as multidrug resistance amongst clinical isolates. This review highlights concerns relating to the use of common antimicrobials as the choice for optimal therapy of common pathogens in Zambia. Our study highlights concern regarding second-line treatment options, such as azithromycin for cholera, in the country have also been highlighted by this study. However, the high rate of antimicrobial resistance does bring into question the testing methods and accuracy of the testing in Zambia as there may have been sample contaminations at certain studies since there were a variety of contradicting results, as seen with penicillin, cephalosporins, monobactams, and carbapenems use for *S. aureus* ranging from 0% resistance to 100% resistance for second line antibiotics (gentamicin, ceftazidime, nalidixic acid and norfloxacin). Similarly, it was noted that resistance has emerged and been detected for *K. pneumonia* and *E. coli*. The results vary widely, however majority shows resistance greater than 50% for both these organisms. These findings demonstrate antibiotic resistance, regardless of testing method, site, year and region, to new and extended spectrum, more efficacious antibiotics.

The most common pathogen identified across the human-animal-environmental sectors was *S. aureus*. *S. aureus* is frequently found on the human skin and is recognized as the main contributor of infections in humans [50]. There are high rates of AMR to gentamycin, ceftazidime, amoxicillin-clavulanic acid, cotrimoxazole, nalidixic acid, norfloxacin and oxacillin found in this study. Gentamycin, ceftazidime (third generation cephalosporin) and the fluoroquinolones (nalidixic acid and norfloxacin) are considered second line for *Staphylococcus spp*. This finding is a serious concern as we see a prominent resistance rate across multiple studies.

Special consideration needs to be addressed in Zambia regarding the surveillance techniques across the several regions. It is imperative to include rural and urban informal settlements, as well as community studies. To prevent an AMR pandemic, it is important to establish surveillance systems that also address and incorporate investigations into the knowledge, attitudes and practices across the human, animal and environmental sectors. It is also recommended to establish systems to map antimicrobial use, resistance profiles and genetics. No known system to address AMR mapping is in place in Zambia at the time of this review. Studies on antifungals, a group that is neglected, should be considered a field of interest. Researchers should be encouraged to collaborate within the human-animal-environmental sectors and conduct studies from a One Health perspective; as well as establish appropriate means to ensure a system can be in place to potentiate future studies on AMR in Zambia.

Our research is one of the first systematic reviews to assess antimicrobial resistance in Zambia. Our results provide critical information that can be used towards policy development and patient management. The different sectors should be more involved and share information to ensure that there is a holistic approach when it comes to combatting AMR.

## Conclusion

To safeguard our current collection of antibiotics it is imperative to address the gaps in AMR diagnostic standardization and reporting; and improve surveillance, stewardship, infection control, and implementations of updated treatment guidelines and monitoring. Overall, this review suggests that improved standardization of antimicrobial susceptibility testing in Zambia could help to better delineate AMR patterns and allow comparisons across different locations and allow tracking of AMR evolution over time.

The findings further emphasize the need to address and implement effective AMR surveillance through continued data sharing, multidisciplinary collaborations, and coordination of all stakeholders—using the One Health Approach. This is essential to understand and manage the AMR national burden especially in Zambia.

## Supplementary Information

**Additional file 1. Table S1:** Detailed findings of antimicrobial resistance rates across the studies included.

## Data Availability

The data and materials of the study will be available from the corresponding author on reasonable request.

## Abbreviations

AJOL: African Journals Online
AMR: Antimicrobial Resistance
BTB: Bovine tuberculosis
CLSI: Clinical and Laboratory Standards Institute
CSE: Centre for Science and Environment
LMIC: Low-middle income countries
MJZ: Medical Journal of Zambia
NAP: National Action Plan
NRTI: Nucleoside reverse transcriptase inhibitors
NNRTI: Non-nucleoside reverse transcriptase inhibitors
PI: Protease inhibitors
PRISMA: Preferred reporting items for systematic reviews and meta-analysis
WHO: World Health Organization
ZNHPI: Zambian National Public Health Institute
