# Supplementary material for: Antimicrobial resistance from a One Health perspective in Zambia: a systematic review
Source: Antimicrob Resist Infect Control. 2023 Mar 3;12:15. doi: 10.1186/s13756-023-01224-0 (PMC9982795; doi:10.1186/s13756-023-01224-0)
Supplement: Supplementary file 1 — Additional file 1. Table S1: Detailed findings of antimicrobial resistance rates across the studies included. [file 13756_2023_1224_MOESM1_ESM.pdf]

						1	2	3	4	5	6	7	8	9	10	11	12	13	14	15	16
						Aminoglycosides					Cephalosporins						Penicillin			Sulfonamides	
						Gentamycin	Streptomycin	Kanamycin	Amikacin	Spectinomyci	Cefpodoxime	Cefotaxime	Cefalexin	Ceftriaxone	Ceftazidime	Cefotixin	Ampicillin	cillin-clavulan	Penicillin	prim-Sulfame	Co-trimoxazol
1	H	Kapatamoyo et al.	Pus Aspirates	*S.aureus*	2010															73%	
2	H	Matundwelo et al.	Pus Swab	*S.aureus*	2016	100%															
3	H	Nagelkerke et al.	Nasal Swab	*S.aureus*	2017																
4	H	Kabwe et al.	Blood	*S.aureus*	2016	50%						0%		33%			33%	100%		67%	
5	H	Chanda et al.	Blood	*S.auerus*											75%						
6	H	Chanda et al.	Urine	*S.auerus*											92%						
7	H	Chanda et al.	Wound Swab	*S.auerus*											57%						100%
8	H	Ziwa et al.	Wound Swab	*S.aureus*	2018	14%			8%		30%	58%		0%	32%		50%		60%		78%
9	A	Youn et al.	Wound Swab	*S.aurues*	2014														64%	36%	
10	A	Schaumburg et al.	Oral Swabs	*S.aurues*	2012														75%	3%	
11	E	Ziwa et al.	Hydrotherapy Bat	*S.aurues*	2018	14%			8%		30%	58%		0%	32%		50%		60%		78%
12	E	Youn et al.	Vet Hospital Facil	*S.aureus*	2014														64%	36%	
13	E	Mwamungule et al.	Doctors Coats	*S.aurues*	2015	50%							50%			68%	75%				68%
14	H	Mainda et al.		*E.coli*	2019	27%		99%	47%		29%	25%		24%	28%		61%	46%			57%
15	H	Chiyangi et al.	Stool	*E.coli*			34%				67%	67%			67%		67%				100%
16	H	Mainda et al.	Stool	*E.coli*	2019	1%					1%						8%			5%	
17	H	Kabwe et al.	Blood	*E.coli*		100%						100%		100%	100%		100%	50%		100%	
18	H	Chanda et al.	Blood	*E.coli*																	67%
19	H	Chanda et al.	Urine	*E.coli*											40%						100%
20	H	Chanda et al.	Wound	*E.coli*											50%						
21	A	Mainda et al.		*E.coli*	2015	1%					2%						6%			4%	
22	E	Songe et al.	Fish Markets	*E.coli*	2016	91%	88%					100%			95%		100%			74%	
23	E	Mwamungule et al.	Doctors Coats	*E.coli*		4%							100%				50%				
24	H	Kabwe et al.	Blood	*Klebsiella*	2016	96%						96%		94%	97%		99%	93%		100%	
25	H	Chanda et al.	Blood	*Klebsiella*																	100%
26	H	Chanda et al.	Urine	*Klebsiella*											69%						0%
27	H	Chanda et al.	Wound Swab	*Klebsiella*																	
28	H	Ziwa et al.	Wound Swab	*Klebsiella*		30%			20%		100%	100%		40%	100%		100%		90%		90%
29	E	Ziwa et al.	Hydrotherapy Bat	*Klebsiella*	2018	30%			20%		100%	100%		40%	100%		100%		90%		90%
30	E	Mwamungule et al.	Doctors Coats	*Klebsiella*		0%							50%								100%
31	H	Matundwelo et al.	Pus Swab	*P.Vulgaris*	2016	63%															
32	H	Nagelkerke et al.	Nasal Swab	*Enterobaciaceae*	2017	78%								52%							
33	H	Chiyangi et al.	Stool	*V.cholerae*	2017	0%						0%				0%	0%				100%
34	H	Mwape et al.		*V.cholerae 2009*													0%			100%	
35	H	Mwape et al.		*V.cholerae 2010*													0%			100%	
36	H	Mwape et al.		*V.cholerae 2016*																	
37	H	Inzaule et al.		*HIV*	2020																
38	H	Bennett et al.		*HIV*	2020																
39	H	Miti et al.	Blood	*HIV*	2020																
40	H	Chanda et al.	Blood	*Citrobacter*																	100%
41	H	Chanda et al.	Urine	*Citrobacter*											75%						100%
42	H	Chanda et al.	Wound Swab	*Citrobacter*																	
43	H	Chanda et al.	Blood	*Coliform*											30%						100%
44	H	Chanda et al.	Urine	*Coliform*											50%						100%
45	H	Chanda et al.	Wound Swab	*Coliform*																	
46	H	Chanda et al.	Blood	*Enterobacter*											0%						100%
47	H	Chanda et al.	Urine	*Enterobacter*											46%						50%
48	H	Chanda et al.	Wound Swab	*Enterobacter*											100%						
49	H	Chanda et al.	Blood	*cnS*											100%						100%
50	H	Chanda et al.	Urine	*cnS*											40%						100%
51	H	Chanda et al.	Wound Swab	*cnS*											75%						100%
52	H	Chanda et al.	Blood	*Proteus*											0%						
53	H	Chanda et al.	Urine	*Proteus*											44%						100%
54	H	Chanda et al.	Wound Swab	*Proteus*											25%						
55	H	Chanda et al.	Blood	*Streptococcus*											100%						
56	H	Chanda et al.	Urine	*Streptococcus*											63%						
57	H	Chanda et al.	Wound Swab	*Streptococcus*											100%						
58	H	Chanda et al.	Blood	*Pseudomonas*																	
59	H	Chanda et al.	Urine	*Pseudomonas*											33%						
60	H	Chanda et al.	Wound Swab	*Pseudomonas*											100%						
61	H	Chanda et al.	Urine	*Yersinia*											100%						
62	H	Kapata et al.	Sputum	*M.tuberculosis*	2015		6%														
63	H	Masenga et al.		*M.tuberculosis*	2017																
64	H	Kapata et al.		*M.tuberculosis*	2013		11%														
65	H	Habeenzu et al.		*M.tuberculosis*																	
66	H	Gill et al.	Nasopharyngeal S	*S.pneumoniae*	2008																65%
67	H	Chiyangi et al.	Stool	*S.Typhi*	2017		100%			75%							100%				100%
68	H	Hendriksen et al.		*S.typhi*	2014		99%										99%	1%	99%		
69	H	Chiyangi et al.	Stool	*S.Paratyphi B*	2017		100%										100%				100%
70	H	Chiyangi et al.	Stool	*NTS*	2017		67%			33%							88%				100%
71	H	Chiyangi et al.	Stool	*S.flexineri*			84%										100%	17%			100%
72	H	Chiyangi et al.	Stool	*S.dysenteriae*													100%				100%
73	H	Chiyangi et al.	Stool	*S.Boydii*													100%				100%
74	H	Chiyangi et al.	Stool	*C.jejuni*													33%				100%
75	A	Youn et al.	Wound Swab	*S.psuedointermedius*		2%													52%	10%	
76	E	Mwamungule et al.	Doctors Coats	*Enterobacter*		50%							75%				25%				
77	E	Mwamungule et al.	Doctors Coats	*Pseudomonas*		25%							100%				75%				

						17	18	19	20	21	22	23	24	25	26	27	28	29	30
						Floroquinolones				B-lactam		Macrolide			Carbapenem	Nitrofuran	Tetracyclines	Chloramphe	Glycopeptide
e						Ciprofloxacin	Nalidixic acid	Ceftazidime	Norfloxacin	Oxacillin		Erythromycin	Azithromycin	Clindamycin	Imipenem	Nitrofurantoin	Tetracycline	Chloramphe	Vancomycin
1	H	**Kapatamoyo et al.**	Pus Aspirates	*S.aureus*	2010	1%				31%		5%					48%	7%	
2	H	**Matundwelo et al.**	Pus Swab	*S.aureus*	2016	32%			22%										
3	H	**Nagelkerke et al.**	Nasal Swab	*S.aureus*	2017					0%									
4	H	**Kabwe et al.**	Blood	*S.aureus*	2016	0%		50%		0%		33%			0%			20%	
5	H	**Chanda et al.**	Blood	*S.auerus*		64%	**100%**		**100%**							0%		38%	
6	H	**Chanda et al.**	Urine	*S.auerus*		65%	92%		75%							38%		37%	
7	H	**Chanda et al.**	Wound Swab	*S.auerus*		40%	**100%**		67%							**100%**		0%	
8	H	**Ziwa et al.**	Wound Swab	*S.aureus*	2018	20%						42%		18%	0%		50%	12%	
9	A	**Youn et al.**	Wound Swab	*S.aurues*	2014												9%		
10	A	**Schaumburg et al.**	Oral Swabs	*S.aureus*	2012					0%		3%		3%			36%		
11	E	**Ziwa et al.**	Hydrotherapy Bat	*S.aurues*	2018	20%						42%		18%	0%		50%	12%	
12	E	**Youn et al.**	Vet Hospital Facil	*S.aurues*	2014												9%		
13	E	**Mwamungule et al.**	Doctors Coats	*S.aurues*	2015	33%			46%			53%		42%			50%	16%	58%
14	H	**Mainda et al.**		*E.coli*	2019	20%											51%	22%	
15	H	**Chiyangi et al.**	Stool	*E.coli*			16%										50%	16%	
16	H	**Mainda et al.**	Stool	*E.coli*	2019	0%											14%		
17	H	**Kabwe et al.**	Blood	*E.coli*		80%						**100%**			0%			60%	
18	H	**Chanda et al.**	Blood	*E.coli*		57%	43%		30%							33%		67%	
19	H	**Chanda et al.**	Urine	*E.coli*		63%	76%	61%	62%							48%		26%	
20	H	**Chanda et al.**	Wound	*E.coli*		**100%**			**100%**							0%			
21	A	**Mainda et al.**		*E.coli*	2015	0%											11%		
22	E	**Songe et al.**	Fish Markets	*E.coli*	2016	95%	93%		91%								91%	81%	
23	E	**Mwamungule et al.**	Doctors Coats	*E.coli*		0%											0%	0%	
24	H	**Kabwe et al.**	Blood	*Klebsiella*	2016	71%						92%			1%			71%	
25	H	**Chanda et al.**	Blood	*Klebsiella*		33%	33%	50%	33%							33%		0%	
26	H	**Chanda et al.**	Urine	*Klebsiella*		68%	56%		40%							75%		44%	
27	H	**Chanda et al.**	Wound Swab	*Klebsiella*		**100%**												**100%**	
28	H	**Ziwa et al.**	Wound Swab	*Klebsiella*		28%						32%		0%	0%		40%	21%	
29	E	**Ziwa et al.**	Hydrotherapy Bat	*Klebsiella*	2018	28%						32%		0%	0%		40%	21%	
30	E	**Mwamungule et al.**	Doctors Coats	*Klebsiella*													50%	50%	0%
31	H	**Matundwelo et al.**	Pus Swab	*P.Vulgaris*	2016	13%			9%										
32	H	**Nagelkerke et al.**	Nasal Swab	*Enterobaciaceae*	2017	60%													
33	H	**Chiyangi et al.**	Stool	*V.cholerae*	2017	27%	**100%**		21%			32%	0%			**100%**	0%	9%	
34	H	**Mwape et al.**		*V.cholerae 2009*			**100%**												
35	H	**Mwape et al.**		*V.cholerae 2010*			**100%**												
36	H	**Mwape et al.**		*V.cholerae 2016*			98%												
37	H	**Inzaule et al.**		*HIV*	2020														
38	H	**Bennett et al.**		*HIV*	2020														
39	H	**Miti et al.**	Blood	*HIV*	2020														
40	H	**Chanda et al.**	Blood	*Citrobacter*		**100%**			**100%**									**100%**	
41	H	**Chanda et al.**	Urine	*Citrobacter*		**100%**	**100%**		**100%**							67%		50%	
42	H	**Chanda et al.**	Wound Swab	*Citrobacter*		0%												0%	
43	H	**Chanda et al.**	Blood	*Coliform*		44%	44%		0%							44%		46%	
44	H	**Chanda et al.**	Urine	*Coliform*		61%	71%		72%							38%		67%	
45	H	**Chanda et al.**	Wound Swab	*Coliform*		50%	83%									75%		33%	
46	H	**Chanda et al.**	Blood	*Enterobacter*		0%	0%									0%		**100%**	
47	H	**Chanda et al.**	Urine	*Enterobacter*		73%	75%		**100%**							67%		88%	
48	H	**Chanda et al.**	Wound Swab	*Enterobacter*		**100%**	**100%**									**100%**		0%	
49	H	**Chanda et al.**	Blood	*cnS*		56%	**100%**									33%		25%	
50	H	**Chanda et al.**	Urine	*cnS*		70%	88%		83%							43%		25%	
51	H	**Chanda et al.**	Wound Swab	*cnS*		67%	**100%**		**100%**							**100%**		0%	
52	H	**Chanda et al.**	Blood	*Proteus*		**100%**	**100%**		**100%**							**100%**		0%	
53	H	**Chanda et al.**	Urine	*Proteus*		63%	78%		50%							67%		83%	
54	H	**Chanda et al.**	Wound Swab	*Proteus*		40%	**100%**		**100%**							**100%**		50%	
55	H	**Chanda et al.**	Blood	*Streptococcus*		67%										**100%**		**100%**	
56	H	**Chanda et al.**	Urine	*Streptococcus*		67%	**100%**		71%							36%		75%	
57	H	**Chanda et al.**	Wound Swab	*Streptococcus*		**100%**										**100%**		**100%**	
58	H	**Chanda et al.**	Blood	*Pseudomonas*												**100%**		**100%**	
59	H	**Chanda et al.**	Urine	*Pseudomonas*		50%	**100%**		67%							50%		**100%**	
60	H	**Chanda et al.**	Wound Swab	*Pseudomonas*		0%	**100%**									**100%**		**100%**	
61	H	**Chanda et al.**	Urine	*Yersinia*		**100%**	60%		**100%**							25%		67%	
62	H	**Kapata et al.**	Sputum	*M.tuberculosis*	2015														
63	H	**Masenga et al.**		*M.tuberculosis*	2017														
64	H	**Kapata et al.**		*M.tuberculosis*	2013														
65	H	**Habeenzu et al.**		*M.tuberculosis*															
66	H	**Gill et al.**	Nasopharyngeal S	*S.pneumoniae*	2008														
67	H	**Chiyangi et al.**	Stool	*S.Typhi*	2017	9%							18%					73%	
68	H	**Hendriksen et al.**		*S.typhi*	2014	4%	3%											83%	
69	H	**Chiyangi et al.**	Stool	*S.Paratyphi B*	2017													**100%**	
70	H	**Chiyangi et al.**	Stool	*NTS*	2017												17%	50%	
71	H	**Chiyangi et al.**	Stool	*S.flexineri*													17%	84%	
72	H	**Chiyangi et al.**	Stool	*S.dysenteriae*													25%	25%	
73	H	**Chiyangi et al.**	Stool	*S.Boydii*														**100%**	
74	H	**Chiyangi et al.**	Stool	*C.jejuni*													33%		
75	A	**Youn et al.**	Wound Swab	*S.psuedointermedius*		4%						10%					25%		
76	E	**Mwamungule et al.**	Doctors Coats	*Enterobacter*		50%											25%	50%	
77	E	**Mwamungule et al.**	Doctors Coats	*Pseudomonas*		25%											25%	25%	

						31	32	33	34	35	36	37	2
							Antivirals			Tuberculosis			
						Colistin	NRTI	NNRTI	Pis	Rifampicin	Isoniazid	Ethambutamo	Streptomycin
1	H	**Kapatamoyo et al.**	Pus Aspirates	*S.aureus*	2010								
2	H	**Matundwelo et al.**	Pus Swab	*S.aureus*	2016								
3	H	**Nagelkerke et al.**	Nasal Swab	*S.aureus*	2017								
4	H	**Kabwe et al.**	Blood	*S.aureus*	2016								
5	H	**Chanda et al.**	Blood	*S.auerus*									
6	H	**Chanda et al.**	Urine	*S.auerus*									
7	H	**Chanda et al.**	Wound Swab	*S.auerus*									
8	H	**Ziwa et al.**	Wound Swab	*S.aureus*	2018								
9	A	**Youn et al.**	Wound Swab	*S.aurues*	2014								
10	A	**Schaumburg et al.**	Oral Swabs	*S.aurues*	2012								
11	E	**Ziwa et al.**	Hydrotherapy Bat	*S.aurues*	2018								
12	E	**Youn et al.**	Vet Hospital Facil	*S.aureus*	2014								
13	E	**Mwamungule et al.**	Doctors Coats	*S.aurues*	2015								
14	H	**Mainda et al.**		*E.coli*	2019								
15	H	**Chiyangi et al.**	Stool	*E.coli*									34%
16	H	**Mainda et al.**	Stool	*E.coli*	2019								
17	H	**Kabwe et al.**	Blood	*E.coli*									
18	H	**Chanda et al.**	Blood	*E.coli*									
19	H	**Chanda et al.**	Urine	*E.coli*									
20	H	**Chanda et al.**	Wound	*E.coli*									
21	A	**Mainda et al.**		*E.coli*	2015								
22	E	**Songe et al.**	Fish Markets	*E.coli*	2016								88%
23	E	**Mwamungule et al.**	Doctors Coats	*E.coli*									
24	H	**Kabwe et al.**	Blood	*Klebsiella*	2016								
25	H	**Chanda et al.**	Blood	*Klebsiella*									
26	H	**Chanda et al.**	Urine	*Klebsiella*									
27	H	**Chanda et al.**	Wound Swab	*Klebsiella*									
28	H	**Ziwa et al.**	Wound Swab	*Klebsiella*									
29	E	**Ziwa et al.**	Hydrotherapy Bat	*Klebsiella*	2018								
30	E	**Mwamungule et al.**	Doctors Coats	*Klebsiella*									
31	H	**Matundwelo et al.**	Pus Swab	*P.Vulgaris*	2016								
32	H	**Nagelkerke et al.**	Nasal Swab	*Enterobaciaceae*	2017								
33	H	**Chiyangi et al.**	Stool	*V.cholerae*	2017								
34	H	**Mwape et al.**		*V.cholerae 2009*									
35	H	**Mwape et al.**		*V.cholerae 2010*									
36	H	**Mwape et al.**		*V.cholerae 2016*									
37	H	**Inzaule et al.**		*HIV*	2020								
38	H	**Bennett et al.**		*HIV*	2020		10%	44%	2%				
39	H	**Miti et al.**	Blood	*HIV*	2020		81%	65%					
40	H	**Chanda et al.**	Blood	*Citrobacter*									
41	H	**Chanda et al.**	Urine	*Citrobacter*									
42	H	**Chanda et al.**	Wound Swab	*Citrobacter*									
43	H	**Chanda et al.**	Blood	*Coliform*									
44	H	**Chanda et al.**	Urine	*Coliform*									
45	H	**Chanda et al.**	Wound Swab	*Coliform*									
46	H	**Chanda et al.**	Blood	*Enterobacter*									
47	H	**Chanda et al.**	Urine	*Enterobacter*									
48	H	**Chanda et al.**	Wound Swab	*Enterobacter*									
49	H	**Chanda et al.**	Blood	*cnS*									
50	H	**Chanda et al.**	Urine	*cnS*									
51	H	**Chanda et al.**	Wound Swab	*cnS*									
52	H	**Chanda et al.**	Blood	*Proteus*									
53	H	**Chanda et al.**	Urine	*Proteus*									
54	H	**Chanda et al.**	Wound Swab	*Proteus*									
55	H	**Chanda et al.**	Blood	*Streptococcus*									
56	H	**Chanda et al.**	Urine	*Streptococcus*									
57	H	**Chanda et al.**	Wound Swab	*Streptococcus*									
58	H	**Chanda et al.**	Blood	*Pseudomonas*									
59	H	**Chanda et al.**	Urine	*Pseudomonas*									
60	H	**Chanda et al.**	Wound Swab	*Pseudomonas*									
61	H	**Chanda et al.**	Urine	*Yersinia*									
62	H	**Kapata et al.**	Sputum	*M.tuberculosis*	2015					1%	6%	0%	6%
63	H	**Masenga et al.**		*M.tuberculosis*	2017					6%			
64	H	**Kapata et al.**		*M.tuberculosis*	2013					5%	13%	4%	11%
65	H	**Habeenzu et al.**		*M.tuberculosis*									
66	H	**Gill et al.**	Nasopharyngeal S	*S.pneumoniae*	2008								
67	H	**Chiyangi et al.**	Stool	*S.Typhi*	2017								**100%**
68	H	**Hendriksen et al.**		*S.typhi*	2014								99%
69	H	**Chiyangi et al.**	Stool	*S.Paratyphi B*	2017								**100%**
70	H	**Chiyangi et al.**	Stool	*NTS*	2017	17%							67%
71	H	**Chiyangi et al.**	Stool	*S.flexineri*									84%
72	H	**Chiyangi et al.**	Stool	*S.dysenteriae*									
73	H	**Chiyangi et al.**	Stool	*S.Boydii*									
74	H	**Chiyangi et al.**	Stool	*C.jejuni*									
75	A	**Youn et al.**	Wound Swab	*S.psuedointermedius*									
76	E	**Mwamungule et al.**	Doctors Coats	*Enterobacter*									
77	E	**Mwamungule et al.**	Doctors Coats	*Pseudomonas*									
